# Our New York Letter

**Published:** 1885-11

**Authors:** Wm. Penny

**Affiliations:** New York


### OUR NEW YORK LETTER.

EDITOR DANIEL'S TEXAS MEDICAL JOURNAL:

*Dear Sir:*

IN my last I gave you a sketch of some of the operations here, and I thought that it might be instructive if not entertaining to your readers, if I should give them the technical methods of antiseptic surgery as actually practiced in the large hospitals of this city. I am aware that the subject is trite, but I also know that it still has an interest for the profession.

The present method contrasts strangely with the old, and let no one be rash enough to attempt to blend them. They are radically antagonistic—the old method was that of rapidity and boldness, the new of painful slowness and caution. It has been related that Deffenbach scouted at exact anatomical knowledge being essential to the surgeon. His motto was "cut and ligate," but this rule is exactly reversed, it is ligate and then cut. For this purpose the most exact anatomical knowledge is required, and but for the work of Claude Bernard the present system of cutting between forceps or ligatures would not have come into use, and the present antiseptic method would have been a failure. That it is not a failure the records of Belleview will fully attest. This hospital, badly located and tainted with the poisons of erysipelas and septicæmia for half a century, now shows a mortality of only five per cent. in the amputations performed there, against fifty per cent. before the introduction of this method.

Two factors then are essential to the successs of antiseptic surgery: First, anesthetics. The use of these gives the time for the nice dissections and the careful toilet of the wound.

Second, absolute cleanliness; and I may add a third: exact anatomical knowledge.

I will now proceed to give you the details of the method employed and the apparatus needed for these operations.

First, what is needed:

First will be sponges; these, if new bleached sponges, will require to be beaten or shaken to get any sand out of them, washed in clear water, then soaked in a solution composed of Labaraque's Solution one part, water ten parts, for half an hour; afterwards washed in either distilled or boiled water until free from the disinfectant.

Second, Ligatures; cat gut is used here almost exclusively. They can be obtained put up ready for use. They are kept in oil of juniper.

Third, Sutures; silver wire, best kept in alcohol as they do not tarnish.

Fourth, Solutions:

A. Bi-chloride of mercury, 1–500
B. Bi-chloride of mercury, 1–240
C. Carbolic acid and glycerine, one part each,
Water 20 parts,

Irrigators; An ordinary fountain syringe and a half gallon pitcher will answer for the general practitioner.

Basins; one to hold instruments, one for sponges, and one to hold bi-chloride sol., 1-2500. Ordinary tin basins will answer, if kept clean.

Instruments; These are made perfectly plain, without maker's name or other device on them; the handles are perfectly smooth, and made of hard rubber. A liberal supply of artery forceps will be required.

Antiseptic Gause—This had better be obtained ready prepared, as it requires a great deal of labor to make it.

Antiseptic Cotton—The borated absorbent cotton is prefered.

Protective sheet, rubber.

Bandages, two and a half to three inches wide, made from ordinary cheese cloth.

Iodoform in powder.

Drainage tubes either rubber or bone.

Now for the technique:

In the place of assuming a case, I will take one from actual practice. Dr. Wyeth, during the last week, removed the tibia from the child and the ulna from a man. I will take the last case, as I saw some years since a similar case in Belleview Hospital. In both cases the outer aspect of the arm was riddled with sinuses. The patient in Belleview was suffering from hectic and septicemia. The house surgeon informed me that amputation had already been discussed. I do not know the fate of this man, but his chances of life were infinitely small. In the case that Dr. Weyth operated on, the disease had not advanced so far, for the reason that they don't wait now. The preparation of the patient consisted in first washing out the sinuses with the bi-chloride solution, 1-2500; next, the washing of the limb with soap and water, then with plain water, to remove all traces of soap; after drying, sponging with sulph. ether, to dessolve any greasy matter that might be in the pores of the skin. The next step was the application of an Esmarch's bandage. The washing was done before etherization. The bandage applied after.

While the patient was being anæsthetized, the surgeon and his assistants had prepared themselves for the operation by removing their coats; they then donned their aprons (for no operation is performed here without an apron on all that assist), then washing themselves with soap and water, with a vigorous use of the nail brush, they, after a copious use of plain water and the usual toweling, proceeded, one and all, to wash their hands in the bi-chloride sol. 1-2500.

The instruments had been washed and placed in a basin filled with the carbolic sol.; sponges in bi-chloride sol. 1-2500.

The strong bichloride solution is used while an Esmarh's bandage is on, and that only to sponge out old sinuses and cheesy joints after being scraped; this is then washed off with the weak solution.

Everything being now ready the operation is proceeded with; the incision is made slowly, each little artery taken up and tied, then the incisions were extended as the bone was found diseased until the elbow joint was opened. The periostome was applied and the periostium separated, all fungous granulations and cheesy matter removed from the joint, the sinuses split into the wound, their surface scraped with a Volkman's spoon, then sponged with the strong bichloride solution, irrigated with the weak solution. the Esmarch removed, all bleeding vessels of any size ligated, the wound elevated, packed with the antiseptic gause which had been dusted with iodoform, the outside of the wound covered with antiseptic gause to the extent of half an inch in thickness. A cover of the rubber protective applied over all. All this required something over two hours time.

I saw this patient on the fourth day after the operation. The highest temperature recorded on his bed card was 102° and that for one evening only. When I saw him it was 99°.

There are some minor variations from this method in some of the hospitals, but it would only be confusing to give them.

Now I have a suggestion to make to any of your readers that wish to try this method: That is, commence with your small operations first, otherwise you will almost certainly fail.

WM. PENNY, M. D.

New York, Nov. 1st, 1885.

